# Effects of the Challenge Initiative’s Community Health Volunteers (CHVs) on Public Sector Service Provision of Family Planning Services in Urban Sindh, Pakistan

**DOI:** 10.3390/ijerph22101528

**Published:** 2025-10-05

**Authors:** Junaid-ur-Rehman Siddiqui, Mansoor Ahmed Veesar, Kashif Manzoor, Irum Imran, Amir Saeed, Faisal Mahar, Saqib Ali Shaikh, Zafar Ali Dehraj, Aaliya Habib, Ghazunfer Abbas, Syed Azizur Rab, Victor Igharo

**Affiliations:** 1 Programs, Greenstar Social Marketing, Karachi 75600, Pakistan; 2 Communication Training Logistics & Supplies, Population Welfare Department Sindh, Karachi 74200, Pakistan; 3 Health Services Karachi, Department of Health Sindh, Karachi 07530, Pakistan; 4 William H. Gates Institute for Population and Reproductive Health, Bloomberg School of Public Health, Johns Hopkins University, Baltimore, MD 21218, USA

**Keywords:** community health volunteers, The Challenge Initiative, urban family planning programs

## Abstract

To counter the high unmet need for family planning in urban areas of Sindh province, Pakistan, Greenstar Social Marketing began implementation of The Challenge Initiative (TCI) in collaboration with the government departments of Population Welfare and Health in eight urban districts of Sindh province. This study aimed to assess the effectiveness of TCI’s Community Health Volunteers (CHVs) on public sector service provision of family planning services in eight urban districts of Sindh province, Pakistan. The Contraceptive Logistics Management Information System (cLMIS) and District Health Information System 2 (DHIS2) were used to obtain monthly contraceptive data from June 2022 to December 2024. CHVs began implementation at different time points in each district, starting from January 2023 to October 2023, when CHVs became operational in all eight districts. Descriptive statistics and two-sample *t*-tests were used for data analysis. CHVs significantly improved family planning service provision, particularly for short- and long-acting methods at the facility level, with greater change observed in Department of Health facilities. This study provides preliminary evidence of the effectiveness of CHVs in increasing public sector service provision of contraceptives, particularly for Department of Health facilities. CHVs bridge the gap between the community and the facility, particularly in areas uncovered by the government’s existing mobilization staff.

## 1. Introduction

There are a total of 15.3 million women of reproductive age (WRA) in Sindh province, Pakistan [1,2]. However, in estimating demand and need for modern contraception, approximately 5.6 million women are excluded from the demand estimates because the Pakistan Demographic and Health Survey (PDHS) only interviews married women of reproductive age regarding fertility and family planning [2,3]. Of the remaining 9.7 million women of reproductive age, all of whom are married women, 49 percent (4.7 million WRA) have a need for modern contraception [2,3]. Approximately 50 percent (2.4 million WRA) of this need has been met by modern contraceptive methods, while the remaining 2.3 million WRA report an unmet need for family planning [2,3]. The method mix for users was heavily tilted toward male condoms and female sterilization, with both methods accounting for 73 percent of all methods in the method mix, with only 6 percent of women reporting usage of long-acting and reversible contraceptives (LARCs), i.e., implants and intrauterine devices (IUDs) [3]. Low uptake of LARCs is particularly counterintuitive as the need for limiting future births accounts for 60 percent of the total need [3]. Furthermore, women with a need for modern family planning who are currently not using a modern method are at risk for unsafe abortions and unintended pregnancies, and are, hence, asked about their future intention to use a family planning method [2,3,4,5]. Of those women reporting an unmet need, 1.3 million women reported intentionality to use a family planning method, while 1 million women were reported as non-intenders [2,3].

Access to contraception is a fundamental human right; therefore, it is imperative that family planning programs in Pakistan expand access and awareness while being grounded in respect for individual autonomy, informed choice, voluntarism, and privacy [6]. Women reporting intention particularly represent low-hanging fruit for family planning organizations working in Sindh, Pakistan, and can easily be counseled to voluntarily become users by approaching them with potential modern contraceptive methods [7,8]. Women reported as non-intenders can be approached to potentially become intenders and users through comprehensive counseling on their family planning options and connecting them with service delivery points [7,8]. Community health workers (CHWs) can play a critical role in counseling and transforming both of these groups into users while counseling women on LARCs who have a need to limit [7,8,9,10,11,12,13].

This study differs from Memon et al.’s (2023), which was a qualitative study conducted in rural Sindh focusing on barriers to family planning adoption [14]. In contrast, this study employed quantitative methods using routine health information system data to assess the effectiveness of CHVs in public sector family planning service provision in urban districts of Sindh province. This paper describes a government-led, cost-effective, and scalable service delivery model to address unmet need for modern contraceptives among women in eight urban districts of Sindh province, Pakistan [11,12,15]. This study presents preliminary evidence about whether the CHV intervention has any effect on family planning uptake in urban public sector facilities. The findings from this study can inform prioritization and scale-up of CHV interventions specifically for improving family planning uptake. This study will additionally explore the effect of CHVs on uptake of short-acting methods, LARCs, permanent methods (PMs), outpatient clients, postpartum family planning uptake (PPFP), and postabortion family planning uptake (PAFP).

### 1.1. Program Description

The Challenge Initiative (TCI) employs a scaling model based on successful practices and lessons from its predecessor, the Urban Reproductive Health Initiative (URHI), which was active in India, Kenya, Nigeria, and Senegal from 2010 to 2015 [11,12,16,17,18,19,20,21,22,23,24]. Since 2022, TCI has operated in Pakistan as a scaling intermediary to help provinces enhance their health systems and implement high-impact interventions (HIIs) in family planning (FP). TCI’s approach involves working closely with provincial and district Departments of Health and Population Welfare that seek to partner with them, provided these governments show political and financial commitment to achieving their family planning objectives, in line with the larger provincial and national FP2030 commitments [25]. The concerned government departments of Health and Population Welfare, in consultation with TCI, decide which HIIs to implement, while TCI offers technical and managerial support, as well as gap funding, to help with planning and building government capacity for effective implementation and coordination. TCI focuses on both expanding the reach of interventions (horizontal scaling) and embedding them into government policies, budgets, and procedures (vertical scaling). Since June 2022, 15 district governments from Islamabad Capital Territory and Punjab and Sindh provinces have collaborated with TCI Pakistan with the aim of increasing additional users for family planning [15]. Moreover, the selected 15 districts account for 28 percent of the total country population and 53 percent of the country’s total urban population [2]. This study focused on the eight urban districts in Sindh with consistent CHV implementation and complete HMIS data. CHV implementation began in the remaining seven districts in late 2024; hence, they were excluded from this study. The TCI program is led globally by the Johns Hopkins Center for Communication Programs in partnership with the Gates Institute for Population and Reproductive Health at the Johns Hopkins Bloomberg School of Public Health, while implementation in Pakistan is led by Greenstar Social Marketing (GSM) [11,12,16,17,18,19,20,21,22,23,24,26]. TCI Pakistan uses various strategies to support provinces in adopting and integrating HIIs into their public health systems. These strategies include promoting advocacy and accountability, strengthening provincial and district leadership and management, providing coaching, and improving data quality and utilization. The TCI HII being focused on in this study is Community Health Volunteers.

#### Community Health Volunteers (CHVs)

In 1994, Pakistan launched the National Program for Family Planning, commonly known as the “Lady Health Workers Program” [27]. This initiative featured two types of health workers: lady health workers (LHWs) and family welfare assistants (FWAs) [27,28]. Lady health workers (LHWs) are employed by the Department of Health and focus on raising awareness and improving access to primary healthcare service providers [29]. Each LHW is tasked with educating 1000 to 1500 people in her community [29]. They conduct door-to-door visits to promote maternal, newborn, and child health (MNCH) and family planning, provide one-on-one counseling, and distribute non-invasive family planning methods like condoms and pills [9,27,30]. They also make referrals to health centers managed by government service providers and other private healthcare facilities [27,28]. Additionally, LHWs receive training in gender-responsive counseling and family planning service delivery [27,29,30,31].

Family welfare assistants (FWAs) have a similar role but are supervised by the Population Welfare Department (PWD) [28,32]. They offer one-on-one counseling to married women of reproductive age (MWRAs), provide short-term methods, promote demand for childbirth spacing and family planning within the community, and refer individuals to service providers [28,32].

There are currently 275 LHWs and 197 FWAs operational across the eight districts in this study (Table 1). For each DOH and PWD facility, there is at least one LHW and FWA attached to that facility. While LHWs were initially focused on family planning service provision, it has been deprioritized by the Department of Health, with most of the LHWs now deputized toward immunization, disease outbreaks, or any other campaign that is a priority issue for the DOH [15,31]. This leaves a large gap in demand generation, particularly for facilities that are located in urban areas and have a greater catchment area. TCI, in collaboration with the respective departments, identified these facilities with “uncovered” areas, and recruited community health volunteers (CHVs) to fill this gap. CHVs were paid a stipend for ten days to conduct household visits and community gatherings to generate demand for contraceptives. Unlike FWAs and LHWs, CHVs did not provide any short-term methods directly; they referred all clients to the facility to which they were attached to. Table 1 below provides a breakdown of LHWs, FWAs, and CHVs by district.

**Table 1:** Distribution of LHWs, FWAs, and CHVs across study districts.

District	LHWs	FWAs	CHVs
Hyderabad	84	34	29
Karachi Central	16	25	23
Karachi East	21	23	25
Karachi Malir	52	16	27
Karachi South	10	32	25
Karachi West	46	19	27
Karachi Korangi	29	29	25
Karachi Keamari	17	19	26
Total	275	197	207

To implement this HII, TCI has laid out five specific steps [33]:

Step 1: Identify and assess CHVs for family planning services

CHVs were women from the community who were already trained in family planning. They often required additional, in-depth training to enhance their skills, ensuring they can deliver high-quality family planning counseling and be effectively integrated into DOH and PWD facilities for client referrals.

Step 2: Provide comprehensive training for CHVs

The identified candidates in each district were consulted to assess their knowledge and comfort level with family planning counseling. Based on this assessment, targeted training was offered to improve their skills in family planning methods, interpersonal communication, counseling, referral processes, and data recording and collection.

Step 3: Equip CHVs with necessary resources

CHVs performed door-to-door visits to generate demand and raise awareness about family planning and birth spacing, and referred clients to their respective clinics for consultation and service provision. TCI ensured that CHVs received the essential equipment and supplies they needed, such as information, education, and communication (IEC) materials in local languages to support outreach and demand-generation efforts.

Step 4: Implement supportive supervision

Bi-monthly and quarterly visits were conducted to review the accuracy of CHVs’ data and provide feedback on their counseling and service delivery. Additionally, monthly meetings were organized at assigned healthcare facilities to evaluate CHVs’ performance, review family planning service data, and address any challenges faced.

Step 5: Utilize CHVs for creative demand generation

CHVs were also trained to lead discussion groups within the community to reinforce family planning information provided through various media like radio programs, TV dramas, and theatre performances.

### 1.2. Hypothesis

This study hypothesized that the CHV intervention in the Department of Health and Population Welfare Department facilities across eight urban districts of Sindh province would increase the average monthly clientele served, particularly for short-acting and long-acting methods, in comparison with the pre-intervention period.

## 2. Materials and Methods

### 2.1. Study Design

This study used a secondary data design using multiple datasets on the number of family planning clients served by intervention districts and facilities.

### 2.2. Data Collection

This study used two Health Management Information Systems (HMISs) to capture service provision at various levels. The contraceptive Logistics Management Information System (cLMIS) provided district-level commodity dispensation data for both DOH and PWD, as well as facility-level dispensation data for PWD; the District Health Information System 2 (DHIS2) was used to obtain facility-level data for DOH facilities [34,35]. Data were obtained from both systems to ascertain the effect of CHVs by department at the district level and the facility level. Since CHVs did not engage in any direct commodity dispensation in the community, all contraceptive uptake driven by CHVs was captured through the HMIS (DOH and PWD district-level dispensation alongside PWD facility-level dispensation in the cLMIS; DOH facility-level dispensation in the DHIS2).

The Contraceptive Logistics Management Information System (cLMIS) was developed for tracking and managing logistics and dispensation of contraceptive commodities [34,36]. Contraceptives supplied throughout the country through the Central Warehouse were recorded manually, resulting in errors and delays on the national scale. To improve accuracy and timeliness, this system was replaced by the online “Contraceptive Logistics Management Information System (cLMIS)” developed in cooperation with the Ministry of National Health Services Regulation and Coordination, the Provincial Departments of Health (DOH) and Population Welfare (PWD), with support of the USAID Deliver Project [35]. To regularize the flow of logistics, the cLMIS was launched in Pakistan in 2010 and gradually spread through most of the country (143 districts) by October 2012 [36]. Since the cLMIS contains data on units dispensed, including data on permanent contraceptive methods, i.e., tubal ligation and vasectomies, it can be used to infer programmatic effects as well [35,36]. The database contains method-specific dispensation data on injectables, emergency contraceptive (EC) pills, condoms, oral contraceptive pills, intrauterine devices (IUDs), implants, tubal ligation, and vasectomies. Aggregated variables were created for short-acting methods (SAMs), LARCs, and permanent methods (PMs).

The cLMIS provides monthly dispensation data from all government facilities in eight TCI intervention districts in Sindh, namely, Hyderabad, Karachi Central, Karachi East, Karachi South, Karachi West, Keamari, Korangi, and Malir. Monthly data were obtained from June 2020 to December 2024; CHVs began implementation at different time points in each district, starting from January 2023 to October 2023, when CHVs were operational in all eight districts.

The Population Welfare Department is the primary custodian of the cLMIS; hence, they enter their facilities’ dispensation data directly into the system, while the Department of Health’s performance is integrated into the cLMIS through their primary MIS called the District Health Information System (DHIS2).

The cumulative district-level consumption for eight districts alongside facility-level clients served for the PWD was extracted from the cLMIS, while DHIS2 was used to extract facility-level clients served for the DOH. In total, 420 facilities (DOH: 258; PWD: 162) were reporting to the district-level dataset without an option to segregate results by facility; in 197 facilities (DOH: 107; PWD: 90), CHVs were operational, which was 47 percent of the total (DOH: 47%; PWD: 56%). Facility-level CHV coverage varied significantly by district, from 27 percent to 83 percent of facilities. Given that CHV coverage was approximately 50% of all facilities, outcomes were analyzed at both the district and facility levels. District-level analyses provided an overall indication of change across the eight districts, while facility-level analyses mitigated dilution from non-intervention facilities. In the facility-level dataset, data on permanent methods were not available; however, data were available on outpatient clients, postabortion family planning clients, and postpartum family planning clients for DOH facilities.

A new variable ‘CHV intervention’ tracing the operationality of CHVs was added to the dataset, marking months without CHV implementation as ‘0’ and months after CHVs began implementation as ‘1’. Since the cLMIS reports on the number of commodities dispensed to clients at health facilities, data were extracted on each contraceptive method separately, and were converted into clients using the couple-years of protection (CYP) conversion factor for condoms (10 condoms = 1 client) and oral contraceptive pills (3 pill cycles = 1 client). This was derived from the USAID benchmark of 120 condoms per year and studies that cite that, generally, clients receive three pill cycles on a monthly visit [37,38]. For all other methods (injectables, IUDs, implants, and sterilization), this study treated one unit dispensed as equal to one client, since these methods were administered or inserted on a per-client basis.

Additionally, data on CHVs’ sociodemographic characteristics were compiled from the programmatic database on CHVs.

### 2.3. Data Analysis

Two-sample *t*-tests were employed as a pragmatic analytic choice suited to inform programmatic decision making [39]. The CHV intervention was rolled out at staggered time points across districts; hence, the post-observation was relatively short in certain districts, limiting the statistical power and robustness of interrupted time-series (ITS) or difference-in-differences (DiD) approaches [40]. Moreover, monthly HMIS data were characterized by reporting inconsistencies, missing data, and fluctuations due to stock-outs, which reduces the stability of highly parametrized models [41]. Monthly client averages, rather than totals, were used to standardize comparisons across facilities and districts with varying reporting volumes, which reduces bias from uneven reporting [42]. This analytical approach was undertaken to balance methodological rigor while ensuring interpretability for policymakers and implementers.

Descriptive statistics were used to analyze the sociodemographic and performance characteristics of the CHVs by department. To infer the effect of CHVs on service provision, two-sample *t*-tests were conducted to compare the monthly average clients of each method and aggregated variables between pre-CHV intervention and post-CHV intervention. This comparison was conducted at the facility-level and the department-level.

## 3. Results

### 3.1. Sociodemographic and Performance Characteristics

A total of 207 female CHVs had been recruited into the program as of July 2024. The mean age of the CHVs was higher in the Department of Health (39.3; SD = 10.5) compared with the Population Welfare Department (33.4; SD = 8.6) (Table 2). Educational attainment across the two departments was largely similar, with more than 40 percent CHVs attaining upper secondary education. Moreover, 38 percent (*n* = 35) of Department of Health CHVs and 47 percent (*n* = 54) of PWD CHVs had attained post-secondary education. Lastly, less than ten percent and less than five percent of CHVs had attained bachelor’s and master’s degrees, respectively. Most CHVs were married in both departments; however, a higher proportion of PWD CHVs were single (36%) compared with DOH CHVs (28%). The distribution of CHVs across districts was largely even, with approximately 23 to 29 CHVs working in each district.

Lastly, in terms of performance, DOH CHVs performed a higher number of average monthly household visits (86.2; SD = 24.9) compared with PWD CHVs (71.1; SD = 33.9). Similarly, DOH CHVs referred an average of 81 women to health facilities (SD = 43.6) each month, while PWD CHVs referred an average of 51 women to health facilities (SD = 36.3). This led to DOH CHVs having a higher referral conversion rate of 94 percent (*n* = 93) compared with PWD CHVs’ conversion rate of 72 percent (*n* = 114).

**Table 2:** Sociodemographic and performance characteristics of CHVs (*n* = 207).

	Department of Health (DOH)		Population Welfare Department (PWD)	
Characteristic	Frequency/Mean	Percentage/SD	Frequency/Mean	Percentage/SD
Age	39.3	10.5	33.4	8.6
Highest level of education attained				
Lower secondary	1	1.1%	0	0.0%
Upper secondary	45	48.4%	49	43.0%
Post-secondary	35	37.6%	54	47.4%
Bachelor’s	9	9.7%	8	7.0%
Master’s	3	3.2%	3	2.6%
Marital status				
Single	26	28.0%	41	36.0%
Married	67	72.0%	72	63.2%
Separated	0	0.00%	1	0.8%
District				
Hyderabad	11	11.8%	18	15.8%
Karachi Central	6	6.4%	17	14.9%
Karachi East	15	16.2%	10	8.7%
Karachi Malir	15	16.2%	12	10.5%
Karachi South	13	14.0%	12	10.5%
Karachi West	12	12.9%	15	13.2%
Karachi Korangi	11	11.8%	15	13.2%
Karachi Keamari	10	10.7%	15	13.2%
Average number of monthly household visits	86.2	25.0	71.1	33.9
Average number of monthly referrals	80.8	43.6	51.1	36.3
Referrals per visit	93	93.7%	114	71.9%

### 3.2. Comparison of Average Monthly Clients

Table 3, Table 4, Table 5, Table 6, Table 7, Table 8 and Table 9 present the results of two-sample *t*-tests that compare the monthly average clients at the district and facility levels before and after the introduction of CHVs. The sample size in Table 3, Table 4, Table 5, Table 6, Table 7, Table 8 and Table 9 represents the number of monthly data points aggregated across districts and facilities. The unit of analysis for Table 3, Table 4, Table 5 and Table 6 is the district (eight districts), and the unit of analysis for Table 7, Table 8 and Table 9 is the facility. The district-level dataset does not allow facility-level disaggregation.

#### 3.2.1. District-Level Assessment

At the district level, this study found a negative effect of CHVs on overall and the PWD’s service provision of SAMs (Table 3). However, for the DOH, this study found that CHV implementation increased service provision for SAMs by 62 percent (*p* < 0.01).

**Table 3:** Average monthly SAM clients in district aggregated by department.

Department	Pre-CHV	*n*	Post-CHV	*n*	Difference	*p*-Value
PWD	4141.4	236	3445.7	167	−17%	<0.001
DOH	1359.0	157	2198.3	168	62%	<0.001
Overall	3029.8	393	2820.1	335	−7%	0.153

Assessing the effect of CHVs on service provision of LARCs, this study found increments across both departments and overall, as well (Table 4). A statistically significant increase of 58 percent (*p* < 0.001) was observed for PWD, while a change of 90 percent was observed for DOH, and an overall 42 percent (*p* < 0.01) increase in LARC service provision was observed.

**Table 4:** Average monthly LARC clients in district aggregated by department.

Department	Pre-CHV	*n*	Post-CHV	*n*	Difference	*p*-Value
PWD	240.1	236	379.4	167	58%	<0.001
DOH	100.3	89	190.3	163	90%	0.163
Overall	201.8	325	286.0	330	42%	0.002

While permanent methods were not a focus for CHVs, minor changes of 13 percent and 17 percent were noted for the PWD and at the overall level, respectively (Table 5). A statistically significant effect of 82 percent (*p* < 0.01) was observed for the DOH.

**Table 5:** Average monthly PM clients in district aggregated by department.

**Department**	**Pre-CHV**	***n***	**Post-CHV**	***n***	**Difference**	***p*-Value**
PWD	85.1	216	96.1	162	13%	0.331
DOH	26.4	32	48.2	22	82%	0.002
Overall	77.6	248	90.4	184	17%	0.203

At the district level, this study found no effect of CHVs at the overall level and on the PWD’s average monthly clientele (Table 6). However, for the DOH, this study found that CHVs improved overall service provision by 73 percent (*p* < 0.001).

**Table 6:** Average monthly clients in district aggregated by department.

Department	Pre-CHV	*n*	Post-CHV	*n*	Difference	*p*-Value
PWD	4243.6	248	3871.9	169	−9%	0.028
DOH	1377.4	162	2389.3	168	73%	<0.001
Overall	3111.1	410	3132.9	337	1%	0.203

#### 3.2.2. Facility-Level Assessment

Since CHVs were attached to facilities, and their successful referrals were recorded as clients under those facilities’ respective reporting systems, facility client volumes, therefore, reflect the effect of CHVs.

At the facility level, this study found statistically significant effects across the board, with improvements of 26 percent (*p* < 0.001), 81 percent (*p* < 0.001), and 27 percent (*p* < 0.001) for the PWD, the DOH, and combined, respectively (Table 7).

**Table 7:** Average monthly SAM clients at facility aggregated by department.

Department	Facilities Reporting	Pre-CHV	*n*	Facilities Reporting	Post-CHV	*n*	Difference	*p*-Value
PWD	79	275.6	1372	88	346.6	871	26%	<0.001
DOH	92	58.2	1857	102	105.2	1583	81%	<0.001
Overall	171	150.5	3229	190	190.8	2454	27%	<0.001

For LARCs, this study found statistically significant effects across the PWD, the DOH, and overall (Table 8). While the magnitude of the change was large, it is pertinent to note that LARC service provision at the facility remained under ten per month at all three levels.

**Table 8:** Average monthly LARC clients at facility aggregated by department.

Department	Facilities Reporting	Pre-CHV	*n*	Facilities Reporting	Post-CHV	*n*	Difference	*p*-Value
PWD	76	4.5	1372	87	9.5	871	110%	<0.001
DOH	57	3.7	1841	87	6.6	1555	77%	<0.001
Overall	133	4.1	3213	174	7.7	2426	88%	<0.001

DOH facilities provide a wider array of healthcare services, notably outpatient and maternal care. While not directly focusing on these services, CHVs had a spillover effect on PPFP and PAFP clientele, increasing them by 73 percent and 91 percent, respectively (Table 9). It is pertinent to note, though, that the magnitude of PAFP clients remained under ten.

**Table 9:** Average monthly clients for outpatient, PPFP, and PAFP at facility aggregated by department.

Department	Facilities Reporting	Pre-CHV	*n*	Facilities Reporting	Post-CHV	*n*	Difference	*p*-Value
OPD	107	2974.2	3574	107	3154.3	2533	6%	0.351
PPFP	81	18.1	1846	93	31.4	1582	73%	<0.001
PAFP	59	2.6	1839	85	5.1	1560	91%	<0.001

## 4. Discussion

The objective of this study was to examine the effect of The Challenge Initiative’s community health volunteers on public sector service provision of modern family planning services in urban Sindh, Pakistan. This is the first study in Pakistan to assess the effect of CHVs on government departments’ service provision.

There are multiple HMISs operating in public facilities of Sindh province, with DOH facilities reporting to the DHIS2 and PWD facilities reporting to the cLMIS [35]. DOH facilities’ data aggregated at the district level are subsequently integrated with the cLMIS through the Application Programming Interface (API). Hence, data were extracted from the cLMIS for both departments, aggregated by district, as well as facility-level data from the PWD, while the DHIS2 was used to extract facility-level data for the DOH. This study conducted a multi-tiered analysis at the district and facility levels to ascertain the effect of CHVs across different levels and systems. It is pertinent to note that there is limited validation of the reported data from the government’s side, which is evident from the wide variation in the number of facilities and districts reporting client volumes in the study period [43,44,45].

This study found that at the district level, the effect of CHVs was diluted at the overall level and for the PWD due to middling facility coverages of 47 percent and 56 percent, respectively. This is largely due to short-acting methods, which account for most of the method mix, suffering from frequent stock-outs due to high demand and weak supply chain management [14,34,35]. In PWD facilities specifically, a decline in SAMs was observed following CHV implementation. This decrease reflects the effect of aforementioned periodic stock-outs in PWD facilities and baseline saturation, given the PWD’s family planning-specific mandate [46]. Contrastingly, this study found significant improvements across the board for the DOH at the district and facility levels. The DOH has a broader mandate, and its staff is often engaged in outreach activities for immunization, dengue, outbreaks, and any other health campaigns. Despite the fact that the Department of Health’s LHW program was initiated with the goal of increasing contraceptive prevalence, the department’s competing priorities have diluted focus on family planning [47,48]. This gap in demand generation and community mobilization was filled by CHVs, leading to pronounced gains.

This study’s results echo findings from previous studies that explored the effectiveness of community health volunteers or workers on improving contraceptive service provision. Studies conducted in Nigeria, Bangladesh, India, and Sri Lanka reported improved contraceptive uptake due to community health worker (CHW) interventions [9,12,49]. A study conducted by the Sukh initiative in Karachi also revealed that CHWs helped in increasing modern contraceptive usage, which contributed to improved CPR [50]. A study with a similar study design and intervention in Ghana reported significant improvements in the government’s service provision of contraceptives [51].

A systematic review found that the CHW intervention leads to greater diversity in the method mix, with an increased number of referrals for long-acting methods [9]. This study reported similar findings, with a significant increase in LARC clients after the introduction of CHVs. This also proved to help in strengthening the health system in low- and middle-income countries (LMICs) as CHVs are often used to overcome the shortage of healthcare staff to contribute to the improvement of primary health services, including but not limited to reproductive health [52].

This study’s findings suggest that CHVs play an important role in overcoming barriers, such as misconceptions, lack of awareness, and access to facilities, to the use of contraceptives, especially for the methods that require counseling and follow-up. This study found a statistically significant increase in LARCs, akin to studies in Ethiopia and Nigeria, which also reported an increase in LARC use with CHW involvement, though they are not directly involved in its provision [53]. The effect of CHWs can be maximized to achieve family planning goals by providing them with the necessary training and supervision [49]. Moreover, unlike prior qualitative work from rural Sindh focused on provider/community perspectives, this study is a quantitative analysis of urban districts assessing the effect of community health volunteers (CHVs) on public sector service provision.

### 4.1. Limitations

This study provides critical insights into the effect of CHVs on government service provision of family planning services; however, there are several limitations inherent to this study’s design. This study utilized secondary data from the cLMIS and the DHIS2, which are the government’s official data sources for assessing family planning performance. Despite this, these systems suffer from limited oversight, inconsistent data entry, quality issues, and under- or over-reporting. This study further acknowledges the effect of improvement in data reporting and concurrent advocacy interventions by TCI, which could also potentially explain the increase in client volumes post-intervention.

There was no comparison group used in this study. The TCI intervention targeted the most urban districts in Sindh province, leaving only rural districts in the province, which do not serve as adequate control sites for the TCI districts. Moreover, CHV coverage varied widely across districts (27–83% of facilities with CHVs operational), leaving few non-CHV facilities for comparison in high-coverage districts. Due to the lack of covariates and controls, causal analyses were not undertaken, as this study’s ability to attribute causal impact was limited due to these factors.

### 4.2. Future Research Directions

Future research should generate causal evidence about the effectiveness of CHVs for improving family planning client volumes by identifying adequate control districts/facilities or using synthetic controls, and using quasi-experimental designs supported by robust analytical methodologies (e.g., difference-in-differences or interrupted time-series analyses). Beyond implementation in Sindh province, studies can focus on the implementation of CHVs in Punjab province, where it would be easier to find control districts/facilities due to the significantly larger population size and network of healthcare facilities. Economic evaluations can be undertaken for cost–benefit analyses and compare the cost-effectiveness of CHVs with LHWs and FWAs in generating client referrals. Lastly, community-based evaluations can assess whether areas with CHVs achieve better health and fertility outcomes compared with areas without CHVs.

## 5. Conclusions

This study aimed to assess the effects of CHVs on public sector family planning service provision in eight urban districts of Sindh province, Pakistan. This study hypothesized that the CHV intervention in the Department of Health and Population Welfare Department facilities across eight urban districts of Sindh province would increase the average monthly clientele served, particularly for short-acting and long-acting methods, compared with the pre-intervention period. The findings support this hypothesis, with significant improvements observed, especially in DOH facilities. The analysis provides preliminary evidence of the effectiveness of CHVs in increasing public sector service provision of contraceptives. Effects were more pronounced in DOH facilities, where CHVs were able to fill gaps in demand generation by generating referrals for family planning services. In contrast, the PWD’s higher baseline and commodity stock-outs limited CHVs’ effect. CHVs bridge the gap between the community and the facility, particularly in areas uncovered by the government’s existing mobilization staff. Government departments and policymakers should review this evidence and focus on institutionalization of CHVs into respective departments’ policies, budgets, and work plans to ensure continuation and sustainability.

## Data Availability

Restrictions apply to the availability of these data. The data were obtained from the government Departments of Population Welfare and Health MISs, and reasonable requests will be considered by the corresponding author, and data will be shared with the permission of the concerned departments.

## Abbreviations

CHV—community health volunteer; cLMIS—Contraceptive Logistics Management Information System; DHIS2—District Health Information System 2; DOH—Department of Health; FP—family planning; HII—high-impact intervention; HMIS—Health Management Information System; LARC—long-acting reversible contraception; OPD—Outpatient Department; PAFP—postabortion family planning; PM—permanent method; PPFP—postpartum family planning; PWD—Population Welfare Department; SAM—short-acting method; SD—standard deviation; TCI—The Challenge Initiative.
